# Relationship between copper and immunity: The potential role of copper in tumor immunity

**DOI:** 10.3389/fonc.2022.1019153

**Published:** 2022-11-07

**Authors:** Fu Cheng, Geng Peng, Yan Lu, Kang Wang, Qinuo Ju, Yongle Ju, Manzhao Ouyang

**Affiliations:** ^1^ Department of Gastrointestinal Surgery, Shunde Hospital, Southern Medical University (The First People’s Hospital of Shunde Foshan), Foshan, Guangdong, China; ^2^ The Second School of Clinical Medicine, Southern Medical University, Guangzhou, Guangdong, China; ^3^ Guangdong Country Garden School, Shunde, Foshan, Guangdong, China

**Keywords:** copper deficiency, CTR-1, IL-2, PD-L1, copper ionophores, copper chelators, tumor immunity

## Abstract

Copper is an essential trace element in an organism, and changes in copper levels *in vivo* often indicate a diseased state. Copper and immunity have been discussed since the last century, with copper deficiency significantly affecting the development and function of the immune system, such as increased host susceptibility to various pathogens, decreased number and impaired function of neutrophils, reduced antibacterial activity of macrophages, decreased proliferation of splenocytes, impaired B cell ability to produce antibodies and impaired function of cytotoxic T lymphocyte and helper T cells. In the past 20 years, some studies have shown that copper ions are related to the development of many tumors, including lung cancer, acute lymphoid leukaemia, multiple myeloma and other tumors, wherein copper ion levels were significantly elevated, and current studies reveal that copper ions are involved in the development, growth and metastasis of tumors through various pathways. Moreover, recent studies have shown that copper ions can regulate the expression of PD-L1, thus, attention should be paid to the important role of copper in tumor immunity. By exploring and studying copper ions and tumor immunity, new insights into tumor immunity could be generated and novel therapeutic approaches to improve the clinical prognosis of patients can be provided.

## Introduction

Copper is an essential trace element in an organism. The redox properties of copper enable it to act as a catalytic cofactor for various enzymes, allowing for protein binding through complexation with cysteine, histidine and methionine, and binding to lower molecular compounds (1, 2). It participates in various redox reactions such as energy metabolism, mitochondrial respiration (cytochrome oxidase (COX)), antioxidation (zinc and copper superoxide dismutase (SOD)), collagen cross-linking (lysine oxidase (LOX)), pigmentation (tyrosinase) and catecholamine biosynthesis (dopamine-b-monooxygenase) (3).

**Figure 1:**
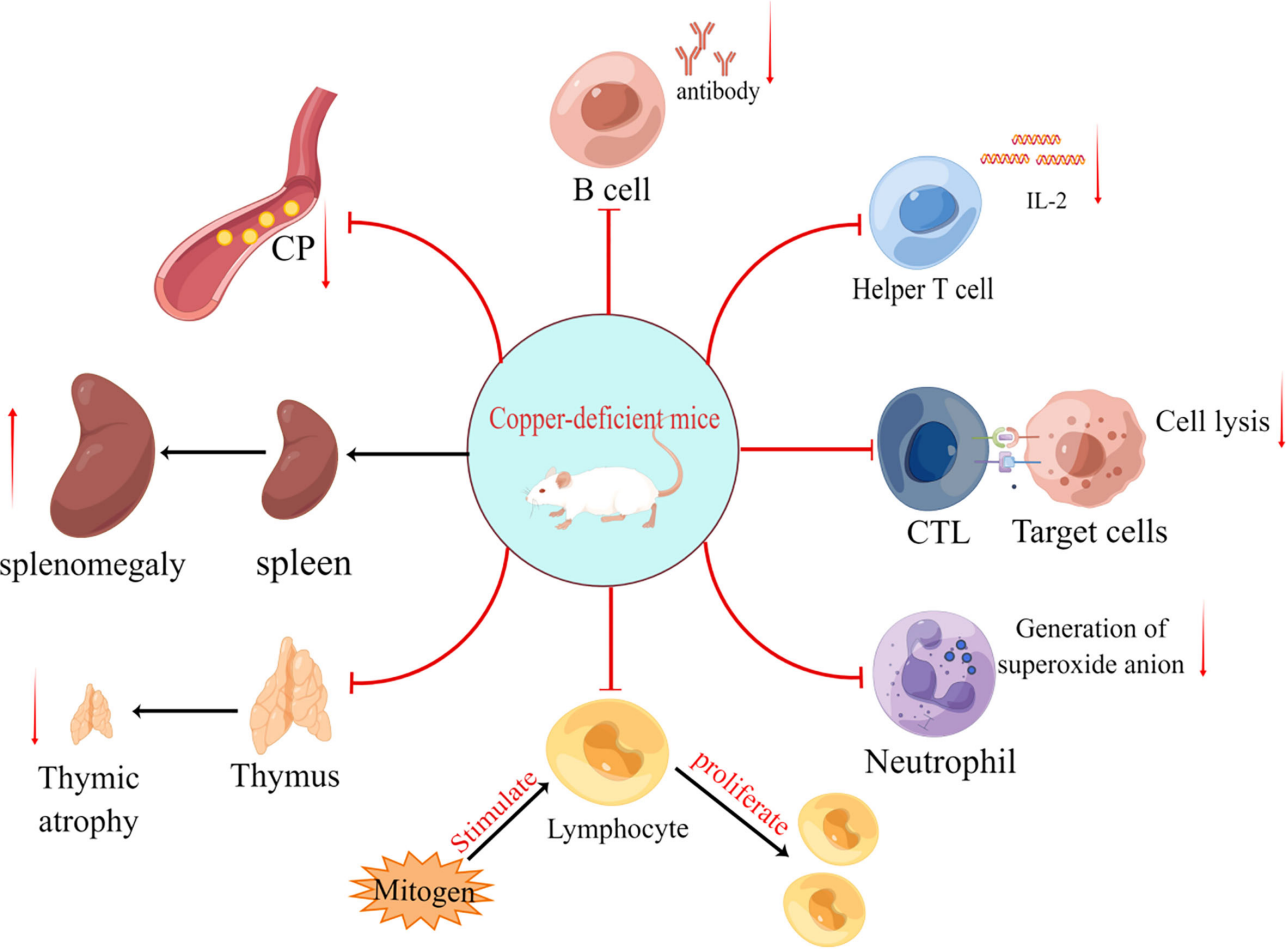
The effects of copper deficiency on mice are divided into eight primary aspects: (i) reduced antibody secretion by B cells; (ii) reduced IL-2 secretion by helper T cells; (iii) reduced target cell killing capacity of cytotoxic T lymphocyte (CTL); (iv) reduced number of neutrophils and production of superoxide anion (O2-); (v) reduced responsiveness of lymphocytes to mitogen stimulation; (vi) thymic atrophy; (vii) splenomegaly and (viii) decreased ceruloplasmin(CP).

Tumorigenesis is inextricably linked to abnormalities of the immune system, and copper ions are considered to be an indispensable trace element in the immune homeostasis of the body. Since the middle of the last century, copper deficiency has been found to significantly affect the development and function of the body’s immune system. Initially, copper deficiency was found to decrease the number of blood neutrophils (4) and impair their anti-bacterial function *via* reducing the production of superoxide anions (5). Subsequently, copper deficiency in various animal models has been found to cause immunosuppression (6–8), leading to the impaired function of immune cells such as B (9) and T cells (10) (**Figure 1**; **Table 1**). Additionally, copper deficiency also affects the development of the immune system in mice (10, 11). These data suggest that copper ions are essential for the proper functioning of the immune system, thereby maybe influencing tumorigenesis and development through immune-related pathways.

**Table 1:** Effect of copper deficiency on the immune system.

	Role classification	Specific role
**Copper deficiency**	Overall	1. Immune system developmental disorders (10, 11) 2. Increases host susceptibility to multiple pathogens (7, 8) 3. Weight loss, thymus reduction, spleen enlargement and cardiomegaly (12) 4. Decreased thymic hormone levels (13), depletion of splenic lymphoid follicles (14), increased acute and delayed inflammatory reactions (15)
	Immune cell proliferating activity	1)Spleen lymphocytes in copper-deficient mice showed a significant decrease in response to LPS/PHA/ConA/PWM stimulation (16)
	Neutrophils	1)Decrease in the number of neutrophils and the production of reactive oxidative species by neutrophils to kill bacteria (4) (5)
	B-cell	1. Increase in the relative percentage of splenic B cells (17) 2. Reduction in the number of antibody-producing B cells and inhibition in the production of antibodies (9, 18)
	T cells	1. The percentage and absolute number of T cells decreased, with the helper (CD4+) T cells showing a significant reduction (17, 19) 2. Suppressing the immunomodulatory function of helper T cells (10) 3. Inhibiting the ability of cytotoxic T lymphocytes to kill target cells (10)
	Cytokine secretion	1. Significant increase in IL-1 secretion in mouse spleen cells (20) 2. Impairment of the synthesis and/or stability of IL-2RNA, thereby significantly downregulating IL-2 synthesis (21) (22) (23)

LPS, lipopolysaccharide; PHA, Phytohaemagglutinin; ConA, concanavalinA; PWM, Pokeweed mitogen.

Alterations in copper levels are often associated with diseases such as Menkes disease and Wilson disease (hepatolenticular degeneration) (24–26), which are characterized by a deficiency or an excess of copper in the body, respectively. The initial studies of Menkes and Wilson diseases laid the foundation for the understanding of intracellular transport and distribution of copper and determining the role of copper and copper metabolism proteins in cell signaling, gene expression and cancer cell proliferation (27). With scientific advances, the abnormal accumulation of copper in cancer cells was found to be an important feature in differentiating them from normal cells. Relevant experimental data showed that several cancer cells maintained trace elements, such as zinc, selenium or iron, at normal levels, but copper levels were significantly elevated (28), such as in stage I multiple myeloma (29), lung cancer (30) and acute lymphoblastic leukaemia (31).

With increasing studies, there is now a certain understanding of the molecular mechanisms of copper involvement in tumor development. Currently, copper has been found to play an important role in the high-affinity copper transporter (CTR-1) (32), LOX (33), hypoxia-inducible factor (HIF-1α) (34), antioxidant protein-1(ATOX-1) (35, 36), RAS-RAF-MEK-ERK signaling pathway (37) (38, 39) and PD-L1 (40), which are associated with tumorigenesis, adaptation to hypoxic environment (41), tumor extracellular matrix construction(ECM), neovascularization, epithelial to mesenchymal tissue transition (EMT) and tumor metastasis. Current studies of these enzymes and pathways identify other gene and protein targets that could be used for cancer therapy.

Current therapeutic strategies using traditional copper-binding molecules, such as copper chelators (ammonium tetrathiomolybdate(TTM)) and copper ionophores (disulfiram (DSF)) (42) (43, 44), have been proposed for cancer treatment and used in clinical trials. Meanwhile, novel copper-binding molecules (e.g., plant-derived copper-binders, autophagy inhibitors, proteasome inhibitors) have gradually become popular research topics due to their antitumor activity and low side effects. Although some of these copper-binding molecules have achieved a certain level of clinical efficacy, certain limitations remain. For example, DSF has not achieved satisfactory results in clinical trials because of its metabolism to diethyldithiocarbamate with low bioavailability *in vivo* (45). Therefore, further clinical trials are needed to prove its efficacy and safety. Owing to the close relationship between copper, immunity and tumor, this review provides new ideas on the use of copper in tumor immunotherapy and the development of better and more effective treatments to improve the prognosis of patients with tumors.

## Relationship between copper and immunity

The recommended daily dietary requirement of copper for a healthy adult is 0.9 mg. Copper is absorbed mainly through the small intestine, enters the liver through the portal vein and is partially excreted through the bile and mostly transported to different tissues and organs by binding to albumin and ceruloplasmin in the blood (3). In the 1950s, researchers found that copper deficiency causes hypocopperemia, hypoceruloplasminemia and neutropenia in infants (4, 6, 46–48) (**Figure 1**). Following this, it was found that copper deficiency leads to impaired superoxide anion production by neutrophils, which kills bacteria (5). Since then, the effect of copper on the immune system has gained increasing research attention.

Macrophages are an important component of the innate immune system and play a key role in the activation and regulation of specific immunological responses through antigen presentation and secretion of cytokines (49, 50), in addition to phagocytosis. In 1991, Bala et al. found that although copper deficiency caused a much larger percentage of macrophages in the spleen of mice than in controls, the absolute number was reduced (19). And Babu et al. discovered that copper deficiency dramatically reduced the production of superoxide anion in macrophages, which in turn affected the ability to kill yeast, Candida, etc., making the organism more susceptible to infection (51). However, studies has been demonstrated that excessive copper supplementation can inhibit macrophage function (52), and so, maintaining copper homeostasis inside and outside the cell is one of the prerequisites for proper macrophage function. While White et al. in 2009 discovered that the effect of copper ions on macrophages appears to be related to the expression of ATP7A in macrophages, they also discovered that pro-inflammatory agents such as LPS or IFN-γ promote copper uptake by macrophages and increase the expression of CTR1 and ATP7A proteins, and demonstrated that the ability of phagocytes to kill pathogens was positively correlated with the expression of ATP7A (53) (54), and consistent with this hypothesis, researchers have identified the process of copper efflux from bacteria *via* copA protein as a defensive mechanism against macrophage killing (55). Tumor-associated macrophages (TAMs) are subtypes of macrophages, which differ from general macrophages in that they exert immunosuppressive effects in the tumor microenvironment by secreting immunosuppressive cytokines (e.g., TGF-β, IL-10) and, while also promote tumor progression and metastasis by secreting growth factors and angiogenic factors (56). Studies targeting TAMs have found that CuNG (a copper chelate) can promote reprogramming of TAMs, promote the secretion of IL-12 and reduce the secretion of TGF-β and IL-10, thus altering their immunosuppressive properties and reactivating the immune response of T cells against tumor cells (57). Although not much research has been done on copper and macrophages, together with the above investigations, we believe that further research and uncovering the mechanism of action between copper and macrophages are feasible and significant for alleviating the immunosuppressive state of TME and enhancing the efficacy of immunotherapy.

In addition to innate immunity, the effect of copper deficiency on acquired immunity has been demonstrated. In 1981, Prohaska et al. observed a reduction in the number of antibody-producing plasma cells in severe and borderline copper-deficient mice, and the extent of this impairment was highly correlated with the degree of hypoceruloplasminemia (9). In the same year, Flynn and Yen demonstrated experimentally that CTL in copper-deficient mice has a reduced ability to target cytolytic cells stimulated by alloantigens and that this defect seems to be associated with the dysfunction of helper T cells (5) (**Figure 1**; **Table 1**). Lukasewycz et al. found that the initiation and maintenance of cell-mediated immunity against leukemic cells were severely impaired in copper-deficient animals (58). Additionally, copper deficiency was also reported to lead to reduced numbers and impaired function of T cells *in vitro* (59) (**Table 1**). Thus, it is clear that copper deficiency affects both humoral and cellular immunity.

The proliferative activity and composition of immune cells are altered in response to copper deficiency. Prohaska et al. demonstrated the reduced responsiveness of splenic lymphocytes in copper-deficient mice models to mitogens such as lipopolysaccharide, phytohemagglutinin and Concanamycin A (16) (**Figure 1**; **Table 1**). The composition of lymphocytes was changed in copper-deficient mice. Furthermore, Lukasewycz et al. (17) analyzed the surface determinant clusters of splenocytes in copper-deficient C58 mice and found that the absolute number and relative percentage of B cells were significantly higher than that in copper-supplemented controls, whereas the relative percentage of Thy 1.2-positive T cells decreased most significantly in the Lyt 1 positive cell (helper T cells) subpopulation (**Table 1**). Additionally, Bala et al. (19) reported a decrease in total splenic monocyte production and the relative and absolute numbers of CD4+ and CD8+ T subpopulations in copper-deficient male rats, however, copper deficiency only increased the relative percentage of splenic B cells and not the absolute numbers. This contrast with the above-mentioned results of Lukasewycz’s experiment could be attributed to the animal model used for the experiment, the experimental method or the apparatus. Another study by Bala et al. demonstrated that dietary supplementation with copper restored the number and function of helper T cells (60). In 1981, Flynn et al. showed that CTL in a copper-deficient medium failed to produce the specific killing of target cells (**Figure 1**; **Table 1**), and which are fully recovered after the addition of T-cell replacement factor (TRF), thus demonstrating the suppressive effect of copper deficiency on helper T cells (10). These experiments demonstrate that copper deficiency leads to a decrease in the number and function of helper T cells. Therefore, copper deficiency has the potential to affect T cells, especially helper T cells, more than B cells.

Copper deficiency affects the production and secretion of immunoreactive substances. In 1981, Prohaska et al. demonstrated that antibody production by splenic plasma cells against sheep erythrocyte antigens was significantly reduced when copper was deficient in cells (9) (**Table 1**). Hamilton et al. also demonstrated that antibody production against sheep erythrocytes was inhibited in copper-deficient mice (P < 0.0001). Contrastingly, antibody production against dinitrophenyl-phenanthroline was not altered by copper deficiency (P = 0.90) (61). In the same year, Koller et al. also demonstrated that copper deficiency affected antibody secretion, resulting in a decrease in plasma antibody titers (18). However, the normal functioning of helper T cells is indispensable for the production of antibodies. Moreover, in Menkes disease, the production of T cells was found to be affected (62). Many of the above experiments have also demonstrated that copper deficiency affects the number and function of helper T cells in experimental animals (10, 17, 19). Furthermore, Lukasewycz et al. (20) found that copper deficiency seemed to have different effects on different cytokines of T cells, such as the overproduction of IL-1 and underproduction of IL-2 (**Figure 1**; **Table 1**). However, the mechanism of copper regulating these cytokines remains unclear. Hopkins et al. in 1997 found that copper deficiency reversibly decreased the amount of IL-2 and IL-2 mRNA in human T lymphocytes (21). In 1999, Hopkins et al. further showed that copper deficiency in human Jurkat T lymphocytes impaired the transcriptional regulation of IL-2 gene expression, resulting in reduced IL-2 production (22) (**Table 1**). Furthermore, in 1992, Bala et al. showed that copper deficiency reversibly restricted DNA synthesis in T lymphocytes by decreasing IL-2 activity, leading to T cell dysfunction (23). Additionally, the reduction in IL-2 synthesis appears to be associated with reduced splenocyte proliferative activity (63). Given these results, it can be speculated that copper deficiency reduces IL-2 synthesis by decreasing IL-2 mRNA synthesis, leading to impaired functions, such as the regulation of other immune cells by helper T cells, the killing capacity of CTL and antibody secretion by B cells.

Interestingly, the effects of copper deficiency on the immune function also vary with gender (64, 65), with male mice appearing to be more susceptible to the effects of copper deficiency (14). However, the mechanisms responsible for this gender difference are unclear.

The occurrence of severe copper deficiency in humans is rare, with borderline copper deficiency state being the most common. Moreover, studies on the effects of copper deficiency on immunity in humans are very few. In 1985, Heresi et al. studied immunoglobulin and neutrophil phagocytosis in children with copper deficiency and found that copper deficiency reversibly impaired neutrophil phagocytosis (66). In 1995, Kelley et al. found that the proliferative capacity of human peripheral blood mononuclear cells and the concentration of IL-2 decreased with a copper-deficient diet, but there was no effect on the peripheral blood cell counts of leukocytes, monocytes, neutrophils, lymphocytes or natural killer cells (67). This result contradicts the significant difference observed in the number and function of neutrophil and lymphocyte subpopulations in animal experiments, which could be attributed to biological differences between animals and humans and the different levels of copper deficiency to which both are subjected. Therefore, the effect of copper deficiency on the human immune system needs further exploration.

## The relationship between copper and tumors

The association between copper and tumors began with the finding that copper levels are elevated in many tumors. In 1965, de Jorge et al. demonstrated an 11-fold increase in copper levels in brain cancer, which was the first study linking tumors to copper levels (68). Subsequently, in 1975, Schwartz highlighted the potential role of copper as a carcinogenic and diagnostic/prognostic marker (69). Meanwhile, studies found elevated copper serum concentrations in various tumors (28), such as stage I multiple myeloma (29), reticulocyte sarcoma, bronchial and laryngeal squamous cell carcinoma, lung cancer (31), leukaemia, lymphoma (70), cervical cancer, breast cancer and gastric cancer (71, 72). In addition to elevated serum copper levels, elevated copper concentrations were reported in the nails and/or hair of patients with tumors, such as breast, prostate and cervical cancers (73). Furthermore, copper deposition in the eye is associated with lung adenocarcinoma (74), multiple myeloma (75) and chronic lymphocytic leukaemia (76). In a clinical study of hematologic malignancies, Kaiafa et al. found that serum copper levels appeared to correlate with disease remission and recurrence (70). Current studies report that elevated copper concentrations in tumor cells are associated with a high expression of CTR-1, however, further investigation is required to determine if there are other factors influencing these copper levels.

Although the specific mechanism of copper involvement in tumor development requires further study, previous reports have identified some of the possible mechanisms of copper involvement in tumor development. CTR-1 is the main transporter protein for the cellular uptake of copper, and researchers have found elevated intracellular copper in various tumors, exhibiting a high expression state of CTR-1. Thus, the hypothesis of CTR-1 promoting tumor development gained attention. Guo et al. (77) in 2021 showed that copper can promote tumorigenesis by activating the phosphatidylinositol 3-kinase (PI3K)-protein kinase B (PKB, also known as AKT) oncogenic signaling pathway and reported that blocking CTR-1 and reducing intracellular copper can inhibit the tumor-promoting effects of this signaling pathway (**Figure 3**). The RAS-RAF-MEK-ERK signaling pathway is another signaling pathway required for cancer formation (37), wherein copper plays a promotional role (38). Copper promotes ERK phosphorylation by binding to MEK1 (copper-binding protein), which leads to RAS/MAPK signaling activation and subsequently to cancer development. Similarly, researchers have found that blocking CTR-1 leads to impaired RAS/MAPK signaling activation, which hinders the oncogenic effects of BRAF/V600E mutation (38, 39) (**Figure 3**). Thus, CTR-1 plays an important role in tumorigenesis, and CTR-1 inhibitors have good therapeutic potential. In addition to participating in tumorigenesis, copper also has a facilitative role in tumor development. Copper promotes the adaptation of cancer cells to the hypoxic environment by reducing degradation (41) and increasing HIF-1α stability (34). It also promotes the construction of the ECM by regulating the activity of LOX (33). HIF-1α and LOX promote tumor development in coordination with each other (78, 79). Furthermore, Eva et al. in 2021 summarized the relationship between copper and tumors and proposed the concept of “cuproplasia”, which is defined as cell proliferation that is regulated by copper (80). It is implicated in many cellular functions, such as mitochondrial respiration, antioxidative defense mechanisms, kinase signaling, autophagy and protein control.

Many tumors stimulate tumor growth through neovascularization (81), which is regulated by angiogenesis-stimulating factors (angiogenin, vascular endothelial growth factor (VEGF), basic fibroblast growth factor (bFGF) and transforming growth factor β (TGFβ)), cytokines (IL-1, -6 and -8)) and angiogenesis-inhibiting factors (vasopressors and endothelial inhibitors) (3). Notably, the activity of many angiogenic factors (calcium-dependent protease (eNOS), angiogenin, platelet-derived growth factor (PDGF)) and their expression (VEGF, fibroblast growth factor (FGF-1) and copper cyanobacteria) are regulated by copper. For example, 1) copper upregulates the expression of several angiogenic genes (e.g., ceruloplasmin, VEGF, FGF-1) (34, 82) by regulating the activation of HIF-1α; 2) copper promotes NO pathway-induced vasodilation by enhancing the enzymatic activity of eNOS (83); 3) copper enhances the function of PDGF and thus promotes smooth muscle cell migration and LOX secretion (84); 4) copper promotes FGF-1 synthesis, thereby promoting vascular endothelial proliferation and ECM formation (83) (**Table 2**). Therefore, copper plays an essential cofactor role in the whole angiogenic signaling cascade and hence copper deficiency can hinder neovascularization (89). Currently, copper chelators are used as neovascularization inhibitors in the clinical treatment of tumors.

**Table 2:** Modulation of angiogenesis-stimulating factor by copper.

Angiogenesis-stimulating factor	The function of copper
VEGF	Increased synthesis of VEGF (82)
PDGF	Promotion of smooth muscle cell migration and LOX secretion (84)
Angiogenin	Copper promotes its interaction with vascular endothelial cells, thereby promoting endothelial cell growth (85, 86)
LOX	Copper acts as an enzyme cofactor for the progression of ECM (87, 88)
HIF-1α	Inhibits degradation and thus promotes the transcription of angiogenesis-related genes (e.g., ceruloplasmin, VEGF) (34)
eNOS	Enhances enzyme activity and induces vasodilation *via* the NO pathway (83)
FGF-1	Copper promotes its synthesis, which in turn promotes vascular endothelial proliferation and ECM formation (85)

**Figure 2:**
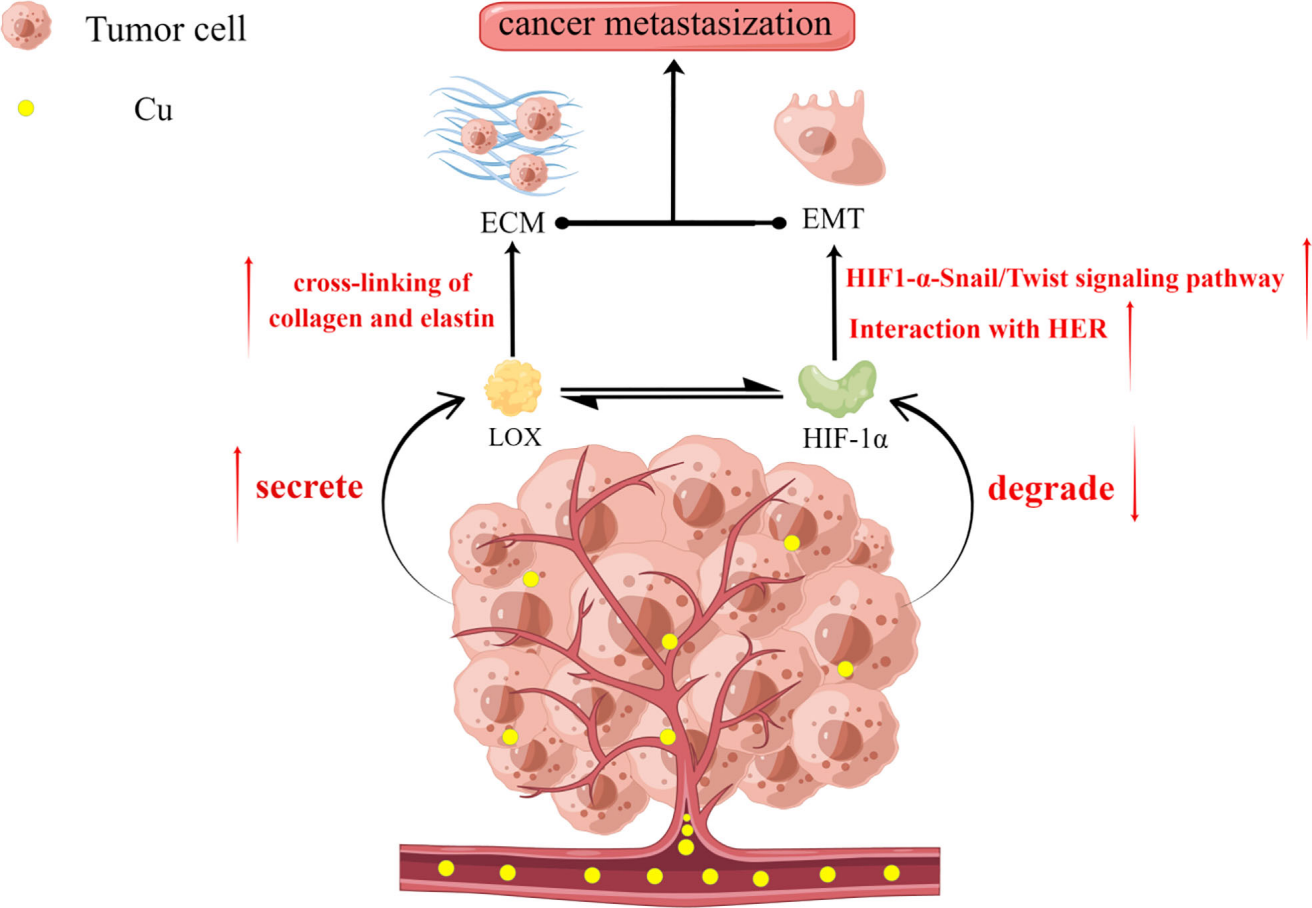
Copper plays an important role in extracellular matrix (ECM) construction and epithelial-mesenchymal transition (EMT), which are two key aspects of tumor metastasis. Copper promotes the secretion of LOX by tumor cells but inhibits the degradation of the structural stability of HIF-1α. There also exists a reciprocal relationship between LOX and HIF-1α. LOX promotes ECM formation by facilitating the cross-linking of collagen and elastin. Increased HIF-1α promotes the adaptation of tumor cells to the hypoxic environment while promoting EMT through interactions with the hypoxia response element (HRE) and the HIF1-α-Snail/Twist signaling pathway. Together, these factors promote the development of tumor metastasis.

Tumor metastasis is an important factor indicating poor patient prognosis. Studies suggest that the two key pathways in tumor metastasis, ECM construction and EMT, are regulated by copper ions (33, 78, 90–92) (**Figure 2**). Cancer cells secrete LOX to promote the cross-linking of collagen and elastin in the ECM (33) and to prepare a suitable environment for the metastasis of tumor cells (93). LOX is a copper-dependent enzyme, and copper ions can induce the secretion of LOX by activating HIF-1α. Moreover, LOX can also promote the synthesis of HIF-1α protein by positive feedback, and the two factors synergize and regulate each other to promote tumor progression (78) (79) (**Figure 2**). LOX also promotes EMT development by stimulating Twist transcription (91). It is also speculated to be an inter-responder of MEMO1, a protein involved in cell migration by regulating the cytoskeleton and forming adhesion sites (94). MEMO1 is a copper-dependent oxidoreductase whose main roles are to produce reactive oxygen species (ROS) that control the redox state of cells and regulate EMT-related transcriptional pathways (95). Copper also contributes to EMT development by activating the interaction between HIF-1α and HRE (96) and the HIF1-α-Snail/Twist signaling pathway (92) to promote the regulation of EMT (**Figure 2**). Some studies have found that the dysregulation of Amyloid Precursor Protein (APP) expression, which contains an extracellular copper-binding domain at the N-terminal end, is also involved in the development of EMT in tumor cells (97–99). Therefore, copper promotes tumor development through multiple pathways and their inhibition is crucial to avoiding poor prognosis due to tumor metastasis.

Current studies have shown that copper is implicated in several key aspects of tumor development. (1) Copper promotes the adaptation of cancer cells to the hypoxic environment by increasing the level of HIF-1α; (2) Tumor angiogenesis is necessary for tumor growth progression, wherein copper is involved in tumor neovascularization by directly interacting with angiogenic factors (VEGF and FGF) (86, 100)and regulating the synthesis of angiogenic molecules (101, 102); (3) Tumor metastasis often implies poor prognosis, and copper promotes tumor metastasis by promoting ECM construction and EMT. Thus, a gradual elucidation of the interrelationship between copper and tumors has been provided, which has therapeutic potential for patients with cancer.

## Copper and tumor immunity-related studies

Copper, immunity and tumors are closely related. Therefore, copper could have a potential regulatory role in tumor immunity. Some studies have reported on the relationship between copper and tumor immunity, such as the reduction of copper in mouse mesothelioma associated with CD4+ T cell infiltration (103), the effect of anti-tumor drug Dp44mT on T cell activity through a copper-related mechanism (104, 105), the apoptosis of bone marrow-derived suppressor cells (MDSCs) and the enhancement of anti-tumor immune responses through selective copper chelation in a drug-resistant tumor model (106) and the downregulation of ACO3 (copper-containing amine oxidase) in lung cancer leading to reduced adherent aggregation of CD4+ cells (107). In a phase II study of high-risk and triple-negative patients with breast cancer, tetrathiomolybdate (TTM) reduced collagen deposition, decreased MDSCs levels and increased CD4+ T cell infiltration in the treated mice (108). In 2020, Voli (40) was the first to propose that copper ions could regulate the expression of the immune checkpoint PD-L1, opening up the possibility and potential of copper ions to participate in tumor therapy by interfering with tumor immunity.

Tumor immune escape is a protective mechanism by which tumor cells protect themselves from the immune system, mainly through the binding of PD-L1, which is overexpressed by tumor cells, to PD-1, which is expressed by lymphocytes, and thereby exerting a negative regulatory effect on T lymphocytes and resulting in diminished cytokine production and cytotoxic effects of immune cells on tumor cells. Through this mechanism, tumor cells can proliferate *in vivo* without the control of the immune system (109). Therapeutic agents targeting this immune checkpoint are currently in clinical application and have greatly contributed to the prognosis of tumor treatment. However, the application of this therapy is currently limited due to the varying reactivity of different tumor cells and the side effects associated with the treatment.

**Figure 3:**
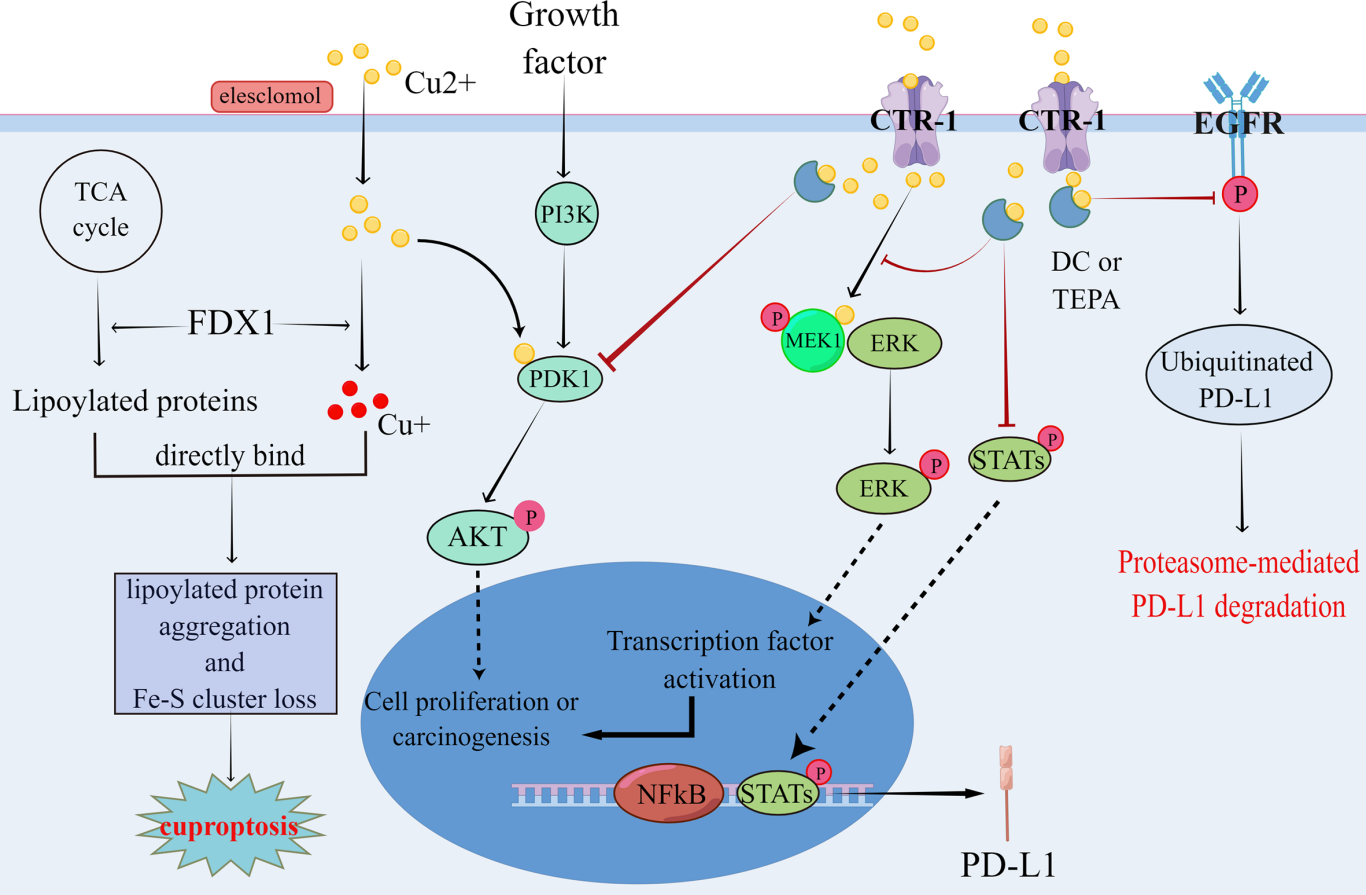
Copper has been shown to promote tumor formation by affecting the regulation of phosphorylation in the PI3K-AKT and RAS-RAF-MEK-ERK signaling pathways, which in turn promotes tumor formation. Meanwhile, copper can regulate PD-L1 expression by both regulating the proteasome-mediated degradation of PD-L1 and affecting the transcription of PD-L1, which leads to tumor immune escape. In contrast, copper chelators can inhibit the aforementioned signaling pathways, thereby suppressing pro-tumor signaling and ameliorating tumor immune escape. cuprotosis is a recently identified copper-dependent mode of cell death, mainly through direct binding of copper to lipid acylated components of the TCA cycle, leading to lipid acyl protein aggregation and loss of iron-sulfur cluster proteins, which in turn leads to cuprotosis due to proteotoxic stress. These theoretical results have contributed to the understanding of the relationship between copper and tumors.

On examining biopsies from 90 patients with neuroblastoma and 90 patients with a brain tumor (including GBM), Voli et al. demonstrated that the three indicators, CTR1/PD-L1/MT1X, have a positive correlation with each other. However, the positive correlation between PD-L1 and CTR1 was limited to tumor tissues. On further exploring how intracellular copper regulates PD-L1 expression, the authors found that Cu and IFN-γ share similar signaling pathways in the regulation of PD-L1 expression and tumor immune response in tumor cells using transcriptome and gene set enrichment analysis. Contrastingly, experiments with copper chelators (DC/TEPA) revealed that DC and TEPA down-regulated PD-L1 expression by inhibiting EGFR signaling and transcriptional activator protein (STAT) phosphorylation signaling pathways (**Figure 3**). Furthermore, they confirmed through several experiments that reducing intracellular copper can inhibit PD-L1 expression at two levels: (1) at the transcriptional level by downregulating the JAK/STAT cellular pathway and reducing PD-L1 mRNA production; (2) at the translational level by promoting the ubiquitination of PD-L1 and facilitating its degradation to downregulate PD-L1 (**Figure 3**). In *in vivo* experiments validated by flow cytometry analysis, the authors confirmed that TEPA and DC increased CD8+/CD4+ T lymphocytes and CD244+ NK cell infiltration. Whole-transcript sequencing of tumor tissues demonstrated that TEPA increased the number of macrophages, CD4+, CD8+ T lymphocytes and NK cells. Furthermore, DC and TEPA reduced IFN-γ (from activated NK cells) mediated PD-L1 upregulation and enhanced NK cell-mediated tumor lysis.

Although several studies and clinical trials have demonstrated that copper chelators are effective in inhibiting tumor growth and angiogenesis, Voli et al. demonstrated for the first time that chelation therapy reduced PD-L1 expression, enhanced anti-tumor immune responses and can be repurposed for immune checkpoint inhibition. Therefore, copper ion-related drugs have great therapeutic potential in tumor treatment.

## Copper related medication

The classical anti-cancer copper-binding molecules are mainly divided into two categories: copper chelators and copper ionophores. Copper chelators act mainly by reducing intracellular copper (110), whereas copper ionophores act by increasing intracellular copper ions, which causes cytotoxic effects through various pathways (111). Furthermore, novel copper-based anti-cancer agents (plant-derived copper-binding molecules, autophagy inhibitors and proteasome inhibitors) are also beginning to emerge (3).

Copper chelators were initially used for the treatment of Wilson’s disease (112). Later, they were tested in tumor treatment and were found to inhibit tumorigenesis, angiogenesis, tumor metastasis and other effects. Common copper chelators include TTM and D-penicillamine (D-pen). TTM is a selective copper chelator that depletes bioavailable intracellular copper and exerts anticancer effects through four pathways: (1) inhibiting angiogenesis through the activation of transcription factors (NF-kB) (113); (2) inducing anti-metastatic activity *via* the inhibition of bone marrow-derived endothelial progenitor cell (EPC) recruitment (114, 115); (3) promoting anti-tumor proliferation activity by reducing ATP production through the inhibition of cytochrome C oxidase function (116); (4) reducing tumor growth in BRAF/V600E transformed cells by decreasing MEK1/2 kinase activity (39). Moreover, TTM is well tolerated in oncology treatment and the resulting side effects such as anemia and neutropenia are reversible (117–119). D-pen was initially a reducing and chelating agent used in the treatment of rheumatoid arthritis, and researchers studying the therapeutic effects of D-pen on arthritis found that it could lead to the reduced chemotaxis of polymorphonuclear leukocytes (120), cause a decrease in antibody titers (121–123) and cause reduced levels of circulating immune complexes (124–126). These results suggest that D-pen could exert immunosuppressive effects. Subsequent experiments have demonstrated that copper salts and ceruloplasmin enhance this immunosuppressive effect, and D-pen was found to inhibit helper T-cell function and antibody production (127–131), which is closely related to the peroxidase enzyme of monocytes (130). D-pen has been found to inhibit LOX secretion and impair collagen cross-linking in tumor therapy (132). It also inhibits tumor angiogenesis by reducing VEGF expression (103). However, some studies have reported serious adverse effects (133). Moreover, D-pen did not improve the survival rate of patients with brain tumors in phase II clinical trials(NCT00003751) (134) (**Table 3**).

**Table 3:** Copper-related drug clinical trials.

cancer	Enrollment	Compound	Phase	Status	ID	Results
Glioblastoma	23	DSF/Cu: 80 mg/1.5 mg p.o. t.i.d 6 months	Phase II	Completed	NCT03034135	Addition of DSF/Cu to TMZ for TMZ-resistant IDH-wild type GBM appears well tolerated but has limited activity for unselected population(161)
Glioblastoma	35	DSF:125/250/375/500mg q.d.	Phase I/II	Active, not recruiting	NCT02715609	Ongoing trial
Glioblastoma	156	CQ: the recommended phase two dose	Phase II	Not yet recruiting	NCT02432417	Ongoing trial
Glioblastoma	40	D-pen: 250 mg ad 2g p.o. q.d. Cu: <0.5 mg/day	Phase II	Completed	NCT00003751	D-pen did not improve survival in patients with glioblastoma multiforme(129)
Lung cancer	60	DSF N/A	Phase II	Recruiting	NCT00312819	The combination regimen of DSF and cisplatin and vinorelbine was well tolerated and appeared to prolong survival in patients with newly diagnosed non- small cell lung cancer(135)
Breast cancer	150	DSF: 200 or 400 mg p.o. q.d. Cu: 2mg p.o. q.d.	Phase II	Recruiting	NCT03323346	Ongoing trial
Breast cancer	28	DSF: 400 mg p.o. q.d. Cu: 2mg p.o. q.d..	Phase II	Recruiting	NCT04265274	Ongoing trial
Breast cancer	50	TTM :Induction period: total 180mg per day Maintenance: total 100 mg per day	Phase II	Active, not recruiting	NCT00195091	TTM has been associated with a significant survival benefit in TNBC patients(162)
Pancreas Cancer	74	DSF: p.o. 1-28 or 1-35 days	Phase I	Recruiting	NCT02671890	Ongoing trial
Germ Cell Tumor	20	DSF: 400 mg q.d. 24 months	Phase II	Recruiting	NCT03950830	Ongoing trial
Melanoma	630	Elesclomol/paclitaxel :213/80 mg/m^2^ i.v. weekly for the first 3 weeks of a 4 weeks cycle	Phase III	Terminated	NCT00522834	The addition of elesclomol to paclitaxel did not significantly improve PFS(139)
Multiple Myeloma	38	DSF/Cu: assigned dose level p.o. b.i.d 28 days	Phase I	Recruiting	NCT04521335	Ongoing trial
Various cancers	58	Elesclomol sodium i.v. over 1 hour on cycle days 1, 8, and 15	Phase II	Completed	NCT00888615	This combination was well tolerated but is unworthy of further investigation based on the proportion responding(140)

DSF, disulfiram; TMZ, temozolomide; IDH, isocitrate dehydrogenase; CQ, chloroquine; D-pen, Penicillamine; TTM, tetrathiomolybdate; TNBC, triple negative breast cancer.

Excessive intracellular concentrations of copper ions are cytotoxic because it leads to the excessive production of ROS and substitution of other metals from binding sites within key proteins (73), causing cytotoxicity. Tsvetkov et al. (140) demonstrated a novel copper-dependent cell death described as the binding of copper ions to the lipid acyl component of the tricarboxylic acid cycle (TCA) in mitochondrial respiration, leading to lipid acylated protein aggregation and iron-sulfur cluster protein downregulation. These result in proteotoxic stress and ultimately cell death, and this copper-dependent cell death is known as “cuproptosis” (**Figure 3**). Although copper ionophores have shown selective anti-cancer activity *in vitro* and mouse models (32, 141, 142), the mechanism of this selectivity remains unclear. Currently, common copper ionophores drugs include DSF, Chloroquine (CQ) and Elesclomol (STA-4783). Increasing the level of intracellular bioavailable copper is a common feature of copper carrier drugs. Many studies have shown that DSF can be used for treating bone metastases in breast cancer (143), ocular melanoma whit liver metastases (144) and non-small cell lung cancer(NCT00312819) (136) (**Table 3**). Additionally, DSF inhibits the proliferation, migration and invasion of hepatocellular carcinoma, especially the nuclear translocation of NF-κB subunit and the expression of Smad4 and leads to the downregulation of Snail and Slug, which inhibit EMT development and hinder tumor metastasis (145). CQ, a derivative of chloroquine, was originally synthesized as an antibacterial agent, but it was found to induce cell death by activating multiple apoptotic pathways in cancer cells (146). The efficacy of CQ was positively correlated with extracellular copper levels (147). Moreover, the results of clinical trials with Elesclomol as a potential copper ionophore-anti-cancer drug were unsatisfactory(NCT00522834, NCT00888615) (138, 139) (**Table 3**). However, subsequent analyses showed that Elesclomol affected tumors that depended on mitochondrial energy production and that FDX1, the gene encoding the target protein of Elesclomol, promotes cuproptosis (148–150). Thus, the identification of the mechanism of cuproptosis could provide more theoretical support for the application of Elesclomol.

Novel copper-based anti-cancer agents have received increasing attention over the past few years. Plant-derived copper-binding molecules (curcumin, Oleuropein-Cu complexes and resveratrol-copper complexes) have been reported to exert anti-cancer effects and increase the anti-tumor activity of known anti-cancer drugs with low side effects. These compounds act as antioxidants, but in the presence of metals such as copper, they act as pro-oxidants that catalyse ROS formation and DNA degradation. For example, curcumin has been experimentally shown to inhibit tumor growth and reduce angiogenesis, and resveratrol-copper complexes have been reported to cause DNA breakage (151). The ubiquitin proteasome pathway (UPP) is responsible for protein targeting and proteolytic degradation and plays a central role in the regulation of cell cycle progression, signal transduction, differentiation, proliferation and apoptosis (152). When traditional copper-binding compounds (i.e. CQ, DSF and TTM) enter cancer cells, they form proteasome inhibitor complexes to affect the proteasome pathway (153). Copper-based compounds have also been developed to target the autophagic process, and the inhibition of autophagic signaling has been demonstrated in human malignant glioma (154) and hepatocellular carcinoma (155).

The deletion of CTR-1 has also been found to be related to platinum anti-cancer drug resistance in tumor cells (156, 157). This could be a breakthrough in tumor resistance to platinum anti-cancer drugs. In human ovarian tumor grafts, the resistance to cisplatin and carboplatin was overcome by co-treatment with selenite, a drug used as a chemotherapeutic adjuvant (158). One possible mechanism to explain this effect could be attributed to the fact that selenite increases the expression of the antioxidant enzyme glutoxigenin 1 (Grx1), which in turn promotes an increase in CTR-1 (159). However, subsequent studies did not support this mechanism, indicating the need for further experimentation.

Copper-related anti-tumor drugs are already used clinically; however, reports on drugs related to copper and tumor immunity remain scarce. Recent studies have demonstrated that copper regulates PD-L1 expression, which could inspire the development of drugs that target tumor immunity.

## Discussion

Tumors are a major class of diseases that endanger human health. In the process of fighting with tumors, human beings have discovered more and more weapons, such as surgical resection, chemotherapy, radiotherapy, immune-targeted therapy, and so on. However, it seems that tumors are also evolving, such as resistance to chemotherapy drugs, post-operative recurrence, immunotherapy side effects and a series of other problems, forcing us to discover more ways to treat tumors. And we believe that there is a close relationship between ion metabolism and tumors, especially the close relationship between copper ions and tumors and immunity, which makes one speculate that new research fields can be opened up for tumor treatment through the study of copper ions and tumor immunity.

Current studies have shown that abnormal levels of copper in the body often indicate a state of disease. And copper deficiency significantly affects the development of the immune system and normal immune function. However, the mechanism of how copper is involved in the regulation of immunity has not been elucidated and further studies are needed. Also copper ions are involved in the regulation of three important tumor properties, namely, infinite proliferation, angiogenesis and metastasis. Excitingly, the finding that copper ions can regulate the expression of the immune checkpoint PD-L1 and increase the tumor infiltration of T and NK cells, as well as cuproptosis, has raised new expectations about the possibility and great potential of copper ions to participate in tumor therapy by intervening in tumor immunity. However, while copper deficiency in normal organisms leads to immunosuppression as previously described, in tumor cells, copper chelators can increase immune cell infiltration and inhibit immune escape by decreasing copper levels. While IL-2 biphasic (160) finding, i.e., it can promote immunity and suppress tumorigenesis in early tumor stages, but in mid- to late-tumor stages, IL-2 signaling promotes 5-hydroxytryptophan (5-HTP) production *via* the STAT5-TPH1 pathway, leading to CD8+ T-cell depletion. Combined with the aforementioned studies showing that copper deficiency inhibits IL-2 production, this seems to explain the paradox to some extent, but the complete answer requires further study.

## Author contributions

MO and YJ are responsible for the concept and design of this study. Material preparation was done by KW. FC and GP drafted the article and QJ and YL revised it critically for important intellectual content. FC and GP retrieved the data and revised the article. FC and GP participated in manuscript writing and editing, who contributed equally to this work. The final manuscript was read and approved by all authors.

## Funding

This study was funded by the Natural Science Foundation of Guangdong Province, China (No. 2022A1515012315), the Beijing Science and Technology Medical Development Foundation (No. KC2021-JX-0186-94),the 2021 Special Innovation Project of Guangdong Provincial Department of Education (No. 2021KTSCX015), In-depth promotion of the innovation-driven assistance project in Foshan City (No. 2021043), the 2018 Foshan City Outstanding Young Medical Talent Training Project (No. 600009), 2020 Shunde District Competition Support Talent Project (no serial number), Southern Medical University Shunde Hospital Scientific Research Startup Plan (No. SRSP2018001), Guangdong Medical Science and Technology Research Fund Project (No. A2019302), the Science and Technology Plan Project of Foshan Science and Technology Bureau (No. 2018AB000683), National Natural Science Foundation of China, National Natural Science Youth Fund Project (No. 81802879), Southern Medical University Scientific Research Startup Plan (No. PY2018N110) and Foshan City’s 13th Five-Year Key Specialty Project (FSGSP2D135051).

## Acknowledgments

We thank Figdraw (www.figdraw.com) for assistance in the pattern drawing.

## Conflict of interest

The authors declare that the research was conducted in the absence of any commercial or financial relationships that could be construed as a potential conflict of interest.

## Publisher’s note

All claims expressed in this article are solely those of the authors and do not necessarily represent those of their affiliated organizations, or those of the publisher, the editors and the reviewers. Any product that may be evaluated in this article, or claim that may be made by its manufacturer, is not guaranteed or endorsed by the publisher.
